# Predicting Delirium Duration in Elderly Hip-Surgery Patients: Does Early Symptom Profile Matter?

**DOI:** 10.1155/2013/962321

**Published:** 2013-02-27

**Authors:** Chantal J. Slor, Joost Witlox, Dimitrios Adamis, David J. Meagher, Tjeerd van der Ploeg, Rene W. M. M. Jansen, Mireille F. M. van Stijn, Alexander P. J. Houdijk, Willem A. van Gool, Piet Eikelenboom, Jos F. M. de Jonghe

**Affiliations:** ^1^Department of Geriatric Medicine, Medical Center Alkmaar, P.O. Box 501, 1800 AM Alkmaar, The Netherlands; ^2^Research and Academic Institute of Athens, 27 Themistokleous Street and Akadimias, 106 77 Athens, Greece; ^3^University Hospital Limerick and Department of Adult Psychiatry, University of Limerick Medical School, Limerick, Ireland; ^4^Medical Center Alkmaar, Pieter van Foreest Institute for Education and Research, 1800 AM Alkmaar, The Netherlands; ^5^Department of Surgery, Medical Center Alkmaar, 1800 AM Alkmaar, The Netherlands; ^6^Department of Neurology, Academic Medical Center, P.O. Box 22660, 1100 DD Amsterdam, The Netherlands

## Abstract

*Background*. Features that may allow early identification of patients at risk of prolonged delirium, and therefore of poorer outcomes, are not well understood. The aim of this study was to determine if preoperative delirium risk factors and delirium symptoms (at onset and clinical symptomatology during the course of delirium) are associated with delirium duration. *Methods*. This study was conducted in prospectively identified cases of incident delirium. We compared patients experiencing delirium of short duration (1 or 2 days) with patients who had more prolonged delirium (≥3 days) with regard to DRS-R-98 (Delirium Rating Scale Revised-98) symptoms on the first delirious day. Delirium symptom profile was evaluated daily during the delirium course. *Results*. In a homogenous population of 51 elderly hip-surgery patients, we found that the severity of individual delirium symptoms on the first day of delirium was not associated with duration of delirium. Preexisting cognitive decline was associated with prolonged delirium. Longitudinal analysis using the generalised estimating equations method (GEE) identified that more severe impairment of long-term memory across the whole delirium episode was associated with longer duration of delirium. *Conclusion*. Preexisting cognitive decline rather than severity of individual delirium symptoms at onset is strongly associated with delirium duration.

## 1. Introduction

Postoperative delirium is a common complication in elderly hip-fracture patients, that is associated with high mortality, cognitive deterioration, and a high rate of subsequent institutionalization [1–3]. Delirium follows a variable course, ranging from a brief transient state to more persistent illness that can evolve into long-term cognitive impairment [4, 5]. Factors that may allow earlier identification of patients who are at risk of more prolonged delirium are not well understood [6, 7]. Although studies over the past decade have improved our understanding of the phenomenology of delirium, little is known about the association between specific delirium symptoms and duration of delirium [8].

Few studies have examined delirium symptoms as a risk factor for an extended duration of the delirious episode. Previous studies have used the Delirium Rating Scale (DRS) [9] to measure the severity of delirium symptoms [10, 11]. Rudberg et al. (1997) found that patients experiencing delirium of a single day's duration did not differ from more persistent (multiple days) cases with regard to individual DRS item scores on the first day of delirium [10]. Conversely, Wada and Yamaguchi (1993), who also used the DRS, found that more severe cognitive impairment, sleep-wake cycle disturbances, and mood lability were associated with longer delirium episodes (>1 week versus ≤1 week) [11]. However, these studies used the original DRS which focuses upon a relatively narrow range of delirium symptoms compared to the revised version (DRS-R-98) and/or did not control for factors such as preexisting cognitive problems, including dementia. This is a significant shortcoming of previous research since dementia may be a predictor of illness duration [12, 13], in addition to being an important risk factor for delirium [14]. 

Most studies that investigated delirium duration restricted delirium monitoring to specific time intervals. The risk of mortality is increased by 11% for every additional 48 hours that delirium persists [15]. This makes it imperative to gain more insight into the determinants of delirium duration. Moreover, frequent (e.g., daily) assessments make it possible to determine that the character of delirium is related to episode duration. 

In this prospective observational study we investigated a homogenous cohort of elderly hip-surgery patients aged 75 or older, who were carefully monitored on a daily basis for the occurrence of delirium. The aim of the present study was to identify patient characteristics that are associated with prolonged delirium and explore how delirium symptomatology evolves over time.

## 2. Methods 

### 2.1. Ethical Considerations

The study was undertaken in accordance with the Declaration of Helsinki and the guidelines on Good Clinical Practice. Approval of the regional research ethics committee was obtained. Patients or their relatives gave fully informed written consent.

### 2.2. Study Design and Objectives

This was a prospective cohort study in elderly hip-fracture patients. Evaluating the relationship between patient characteristics and delirium was a prespecified aim of this study. 

Patient characteristics and risk factors for delirium were assessed preoperatively. Presence and severity of delirium were assessed daily. Since all participants were at high risk for delirium (i.e., age 75 years or older, and acute hospital admission), all patients received routine care with prophylactic treatment of 0.5 mg haloperidol, three times daily, from time of admission until postoperative day three, unless contraindications regarding its use were present [16].

We investigated the association between delirium symptoms on the first delirious day, with subsequent duration of the delirious episode. We compared incident delirium cases experiencing short delirium episodes (1 or 2 days) with patients who experienced more prolonged delirium (≥3 days). Thereafter, we investigated the association between delirium symptom profile over time and duration in days until recovery. For this longitudinal analysis, data on DRS-R-98 item scores gathered over all days of active delirium was included. 

### 2.3. Participants

The study was conducted in a series of consecutively admitted elderly hip-fracture patients to a teaching hospital in Alkmaar, The Netherlands. Eligibility was checked for all patients 75 years and older admitted for primary surgical repair of hip fracture. From March 2008 to March 2009, 192 hip-fracture patients were eligible, and they fulfilled criteria for participation and provided consent. A subgroup of this study cohort, 122 patients, also participated in a clinical trial that compared the effectiveness of taurine versus placebo in reducing morbidity and one-year mortality in elderly hip fracture patients (Clinicaltrials.gov; registration number NCT00497978; this project has been the subject of a previous report [17]). The 122 patients who participated in the RCT were younger compared to the rest of the 192 eligible patients. 

Patients were ineligible to participate in the study if they had no surgery, had a malignancy, had a previous hip fracture on the identical side, were in contact isolation, incapable of participating in interviews (language barrier, aphasia, and coma), had no acute trauma, were transferred to another hospital, or received a total hip prosthesis. 

For the current analysis we also excluded cases who died during hospitalization, were already delirious before surgery or could not be allocated to one of the duration groups according to the definition of recovery. The people who died during admission were more often male, had a history of previous delirium, and were more dependent in their activities of daily living compared to the rest of the 192 eligible patients. Preoperative delirium cases were excluded because we focused upon a well-defined homogeneous group of incident delirium, and the presence of preoperative delirium includes cases where delirium may have contributed to falls and need for subsequent hip-fracture surgery. Patients with no data available on the two days after the last delirious day could not be allocated to one of the duration groups. In this instance we could not define the exact count of delirious days according to the definition used for recovery. 

### 2.4. Measurements and Procedures

#### 2.4.1. Baseline Assessment

Baseline assessment was completed within 12 hours of admission and prior to surgery. This comprised delirium assessment, patient and proxy interviews and questionnaires, and inspection of the medical record to assess for risk factors for delirium. Preoperative cognitive functioning was assessed with the Mini-Mental State Examination (MMSE) on a scale of 0 to 30 with scores lower than 24 indicating cognitive impairment [18]. Prefracture cognitive decline was estimated with the short version of the Informant Questionnaire on Cognitive Decline in the Elderly (IQCODE-N), scored by a close relative or caregiver. This measures preexisting cognitive decline during the past 10 years on a scale of 16 (improvement) to 70 (decline) [19]. A total score higher than 57 (i.e., a mean item-score higher than 3.6) indicates cognitive decline [20]. For the IQCODE-N proxies were asked to describe the patient's condition a week before the fracture as to determine function unbiased by the event of hip fracture itself or any acute or subacute event leading to hip fracture. Burden of illness included the number and type of medical comorbidities and medications before hospital admission. Demographic factors included age and gender. Data on medication was collected as part of the prospective data collection and checked again afterwards by medical record review. We also reviewed medical records to document the Acute Physiology Age and Chronic Health Examination (APACHE II) score (range of 0 (no acute health problems) to 70 (severe acute health problems)) [21]. Functional status comprised prefracture living arrangement, visual acuity, activities of daily living (ADL), and instrumental activities of daily living (IADL). Visual acuity was assessed with the standardized Snellen test for visual impairment [22], and visual impairment was defined as binocular near vision, after correction, worse than 20/70. Prefracture ADL functioning was determined with the Barthel Index (BI) which is scored by a close relative or caregiver on a scale from 0 (dependence) to 20 (independence) [23]. IADL was also assessed by a close relative or caregiver on the Lawton IADL scale with a range of 8 (no disability) to 31 (severe disability) [24].

#### 2.4.2. Outcome

The primary outcome was duration of delirium. The highly fluctuating nature of delirium makes for problems in reliably defining recovery, and therefore a standard definition is lacking [25]. We followed a conservative approach to define recovery of delirium as two subsequent days without delirium according to the Confusion Assessment Method (CAM) [26]. For some analyses (specified in what follows) delirium duration was used as a continuous variable, whereas we also used a dichotomy with incident cases who were delirious for 1 or 2 days labeled as “short delirium” with the remaining cases who were delirious for three days or more, labeled as prolonged delirium. A single day without delirium but followed by further delirium was considered part of the delirium episode. 

Delirium was defined according to the Confusion Assessment Method (CAM) and validated with a diagnosis based on DSM IV criteria [26, 27]. The CAM consists of acute onset and fluctuating course of cognitive function, inattention, and either disorganized thinking and/or altered level of consciousness. Delirium severity was measured using the Delirium Rating Scale Revised-98 (DRS-R-98), a 16-item rating scale comprised of thirteen severity items and 3 diagnostic items. The item scores have range of 0 (no severity) to 3 (maximum severity). Possible total severity scores have range of 0 (no severity) to 39 (maximum severity) [28]. Presence and severity of delirium were assessed within 12 hours after admission and before surgery and continued daily after delirium onset or until the fifth postoperative day for delirium onset. Delirium usually presents itself within the first few days after surgery, if delirium onset is after this time frame it is mostly caused by secondary complications (e.g., urinary tract infection) [29–34]. The CAM and DRS-R-98 rating were based on all available information, collected by trained research assistants, including (i) brief formal cognitive testing with the MMSE, (ii) patient and hospital staff interviews, and (iii) scrutiny of the medical and nursing records. 

### 2.5. Data Analysis

Data analysis was performed using SPSS for Windows, version 19 (SPSS, Inc., Chicago, Il). Comparisons of group characteristics were made using chi-square or Fisher's exact test for differences in proportions, *t*-testing for differences in means, and nonparametric tests for rank differences. 

The univariate significant baseline variables between the short and prolonged delirium group were analyzed with binary logistic regression using the backward Wald method, in order to select the control variables for the first and second part of the research question.

For the first research question (the prediction of short versus prolonged delirium duration by the initial severity of DRS-R-98 items and baseline characteristics) binary logistic regression with the backward Wald method was used. Delirium duration was the binary-dependent variable (short (≤2 days) versus long (≥3 days)). 

At first we applied a logistic regression model with only the scores (range 0 to 3) on the 13 DRS-R-98 severity items on the first day of delirium. Afterwards the same model was repeated but including the covariates: age, sex, and prior cognitive decline (IQCODE > 3.6). The variables were checked for collinearity (collinearity statistics, tolerance, and variance inflation factor (VIF) were performed; all variables entered into the model had a VIF less than 10 and Tolerance more than 0.1).

 For the second research question (clinical symptomatology during the course of delirium) a generalised estimating equations model was used to analyze longitudinal data for patterns of individual items from the DRS-R-98 (items 1–13) between cases with different delirium duration until recovery (range from 1 through 9 days). All available DRS-R-98 item scores (1–13) from first day of delirium until the defined end of the delirium episode were included as independent variables. The continuous dependent variable was duration, measured as the sum of the delirium days from the first day of delirium until the end of delirium. Because it is factually a count variable, following a Poisson distribution, we treated this as such. The GEE method takes into account the fact that observations within a subject are correlated and estimates the population average across time. All scale items were included in each analysis although only those that were significantly different are shown in the results tables.

Results were classified as significant if the *P* value was less than 0.05.

## 3. Results

**Figure 1:**
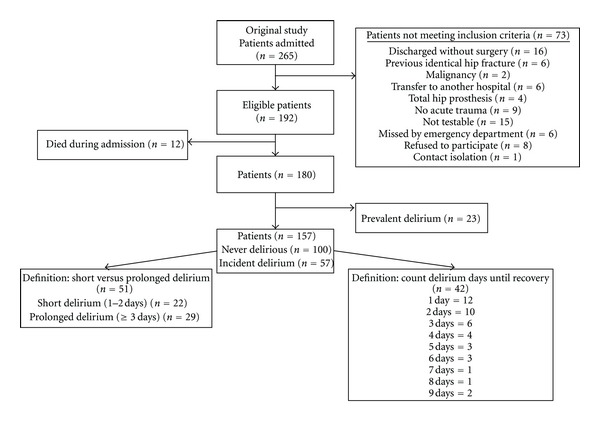
Flow diagram of the study.

After excluding ineligible patients (*n* = 73), patients who died in hospital (*n* = 12), and prevalent cases (*n* = 23) there were 57/157 (36.3%) incident delirium cases (Figure 1). Six cases were excluded, since they could not be defined with certainty as belonging to the short or prolonged delirium group because of missing data. The second research question involved exclusion of another 8 cases because of missing data that impeded determining exact duration of delirium according to our definition of recovery. Treatment (taurine or placebo) had no effect on daily CAM diagnosis, DRS-R-98 total scores, and delirium duration, so this was not entered as a control variable in further analysis. Logistic regression analysis with baseline characteristics identified IQCODE score > 3.6 as the only significant factor, so this was entered as a control variable in further analysis. 

The average age of the 13 male and 38 female patients was 85.1 ± 5.4 (mean ± standard deviation). A total of 22/51 cases (43.1%) had short delirium (1 or 2 days) and 29/51 cases (56.9%) had prolonged delirium (≥3 days). Within the prolonged delirium 20/28 cases could be further defined with regard to exact duration (3 days: *n* = 6; 4 days; *n* = 4; 5 days, *n* = 3; 6 days: *n* = 3; 7 days: *n* = 1; 8 days: *n* = 1 and 9 days: *n* = 2). A significantly greater (*P* = 0.003) proportion of patients within the prolonged delirium group (26/29 cases: 89.7%) compared to the short delirium group 11/22 cases (50%) had an IQCODE > 3.6. A further comparison of the short and prolonged delirium group on other variables is depicted in Table 1. The use (yes or no) of medication classes (sedative hypnotics, antipsychotics, opioids, beta-blocking agents, antidepressants, antihistamines for systemic use, antiparkinson agents, corticosteroids for systemic use, nonsteroidal anti-inflammatory agents, antiepileptics, diuretics, and H_2_-antagonists did not differ significantly between the short and prolonged delirium group.

Table 2 shows a descriptive analysis of the presence (score ≥1) of delirium symptoms within the short and prolonged delirium group on the first day. Disturbed orientation and attention were prominent features in both groups. Only visuospatial functioning differed significantly (OR 5.3, 95% CI 1.28–21.57, *P* = 0.02).

**Figure 2:**
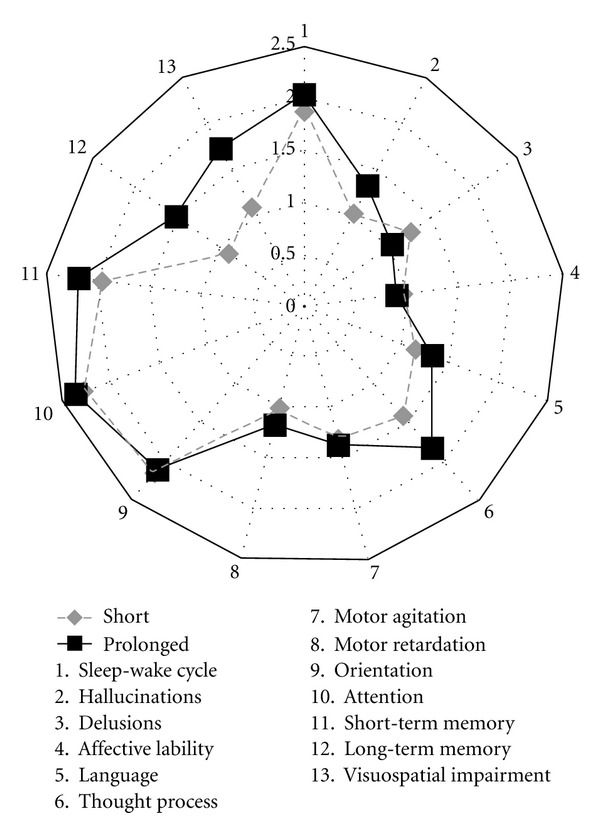
Mean DRS-R-98 item scores for short (1-2 days) versus prolonged (≥3 days) delirium on the first delirious day.

Figure 2 displays the mean scores on DRS-R-98 items on the first day of delirium within the short and prolonged delirium groups. None of the individual item scores differed significantly between the groups.

Logistic regression analysis indicated that more severe motor retardation on the DRS-R-98 was associated with prolonged delirium (OR 1.88, 95% CI 1.03–3.42, *P* = 0.04). The model's *R* ^2^ (Nagelkerke) was 0.16, and percentage of correctly classified patients was 61.5% (1-2 days: 0%, ≥3 days: 100%).

After controlling for time-invariant variables (gender, age, and preexistent cognitive decline) none of the DRS-R-98 items on the first day of delirium were associated with delirium duration. Only preexistent cognitive decline (IQCODE > 3.6) was associated with prolonged delirium (OR 0.1, 95% CI 0.02–0.61, *P* = 0.01). The model's *R* ^2^ (Nagelkerke) was 0.24, and the overall percentage of correctly classified patients was 74.4% (1-2 days: 46.7%, ≥3 days: 91, 7%).

The longitudinal analysis with data on DRS-R-98 item scores gathered over all the delirium days gave the final most parsimonious GEE model (113 observations, 38 patients included) that is shown in Table 3. A higher score on long-term memory (DRS-R-98 item 12) was associated with a longer duration of delirium until recovery considering all assessments within the delirium episode. 

## 4. Discussion

This study is one of the few to describe the predictive value of delirium symptomatology in the early phase of the delirium episode for subsequent duration. In a homogenous population of elderly hip-surgery patients, we found that the severity of individual delirium symptoms at the first day of delirium was not associated with short or prolonged delirium. Initially motor retardation was identified as a predictor for longer delirium duration (≥3 days), but when controlling for gender, age, and preexisting cognitive decline, only preexisting cognitive impairment was associated with prolonged delirium. In addition more severe impairment of long-term memory (as it was also measured with DRS-98 R item 12) across the whole delirium episode was associated with longer duration of delirium. 

Preexisting cognitive impairment is thought to be more common in hypoactive delirium, although there is quite limited data to support this observation [35]. Our data indicate that the observed relationship between relatively hypoactive clinical profile (as measured on item 8 of the DRS-R98) and more prolonged delirium is confounded by the relationship between motor retardation and preexisting cognitive impairment. This observation supports clinical experience where delirium superimposed on dementia is more likely to be hypoactive and resolves more slowly. 

There have been few studies investigating the predictive value of delirium symptoms on the first day of delirium, and findings have been inconsistent. Rudberg et al. (1997) determined the duration within a mixed sample of 64 general medical and surgical patients who were found to have delirium [10]. Similar to our findings, there was no difference between delirium lasting a single day versus that of more prolonged cases in relation to individual delirium symptoms. They did find that the multiple day cases had higher DRS total scores on the first day. In contrast, Wada and Yamaguchi [11] found that poor cognitive status, sleep-wake cycle disturbances, and mood lability were associated with delirium lasting more than a week. However, in our study item 12 (long term memory) was the only predictive item from DRS-R-98 for delirium duration such that participants with more severe long-term memory problems experienced more prolonged delirium. However, the two previously mentioned studies measured symptoms with the original 10-item DRS, which includes a more restricted range of symptoms than the revised DRS-R-98 which captures a wider range of cognitive and neuropsychiatric disturbances that occur in delirium and is widely used in the assessment of delirium severity and in phenomenological studies. In addition, both patient populations were highly heterogeneous and not only limited to only postoperative delirium and focused only on univariate analysis without controlling for confounding factors, like preexisting cognitive decline. 

The study by Wada and Yamaguchi described delirium duration according to a general category (≤1 week versus >1 week) [11]. However, we found that almost half of the delirious patients experienced a delirium episode of 1 to 2 days. Rudberg et al. also found a high percentage of cases (69%) with a single day of delirium in their sample [10]. Recent work has highlighted the impact of short periods of delirium upon outcomes and emphasises the importance of daily assessments in studies of delirium [15].

Our work includes some significant strengths that include the use of daily measurements with the DRS-R-98. Moreover, given the challenges in longitudinal studies of handling the effects of dropouts, interdependence of ratings across visits within patients, and individual patient variability in delirium severity over time, we used the GEE modeling method because it manages these issues in longitudinal datasets and is therefore particularly suited to investigating the course of delirium, considering its fluctuating nature. 

Preexisting cognitive decline is thought to be associated with more prolonged delirium. It has been postulated that this reflects the effects of uncontrolled neuroinflammation contributing to delirium symptoms [36]. Experimental findings and neuropathological observations suggest that activation of microglia is pivotal for mediation of the acute behavioural and cognitive effects of systemic inflammation [36]. A mild systemic inflammatory response suffices to increase the production of proinflammatory cytokines within the brain when microglia are already “primed” by chronic pathologic events as chronic neurodegeneration or advanced age [37]. After hip surgery the release of pro-inflammatory cytokines as a consequence of fracture and surgery induces a systemic inflammatory response. Since inflammatory markers have been shown to be elevated in dementia as well as MCI [38–40], it follows that preexisting cognitive impairment might not only increase the chance of developing delirium but also prolong it. 

This work also has some limitations. The present study was naturalistic in design. Patients received optimal care, which incorporates extensive general geriatric care and haloperidol as prophylactic interventions in high risk patients and delirium treatment according to study site protocol. Both are known to impact positively upon the course of delirium. Although we cannot exclude the effect haloperidol might have had on motor symptom profile in this population, similar longitudinal work in a palliative care setting suggests a limited relationship between motor activity and use of antipsychotic agents [41]. Furthermore, since all patients in our sample were of high risk for delirium and thus all received haloperidol prophylaxis, any likely effects would be likely similar for all the patients in our sample. Although a subsample of the patients participated in a clinical trial, analysis showed that study treatment (taurine/placebo) did not have effect on study outcomes. Also, delirium treatment was delivered according to a standard protocol, and this did not differ between the sample participating in the clinical trial and the naturalistic cohort. 

The exclusion of participants who could not be classified regarding the duration of delirium episode might reduce the strength of this study. This study is not the first to define two consecutive negative delirium assessments as resolution of the delirium episode [42]. However, to allow for greater confidence, we repeated the analysis twice. First, the analysis was repeated with the excluded patients added to the short delirium group, because we had data of at least 1 or 2 delirious days. Second, the prolonged delirium group was limited to patients who could be exactly defined according to duration of the delirium episode in exact count of days, similar to the short delirium group. This did not change the results evident in the initial analysis. 

 This study excluded preoperative delirium cases and focused upon incident delirium cases, a well-defined and homogeneous group of elderly hip-fracture patients. Preoperative delirium cases might have experienced hip fracture because of their confusion and subsequent other causes. Lee et al. (2011) demonstrated that delirium duration can last as long as 4 weeks or longer [13]. The main cause of this prolonged delirium was preoperative delirium. The duration of delirium has been noted to be shorter, suggesting that preoperative delirium may include a different group who warrants separate study. 

The sample size was relatively small as it was limited to incident cases of delirium. The main finding that cognition rather than delirium profile is associated with delirium duration was demonstrated by two separate methods. The GEE method is an innovative statistical analysis used for longitudinal data analysis, and the small sample size is less important with this analysis because we have a relative large number of observations due to the use of daily assessments. 

In conclusion, this study explores the relationship between baseline status and early symptoms of delirium with delirium duration in a homogenous population using validated measurement scales. Preexisting cognitive decline, a concept intertwined with dementia, rather than specific delirium symptoms, was the principal predictor of delirium duration. 

## Figures and Tables

**Table 1:** Baseline clinical and demographic characteristics of patients in the short and prolonged delirium group.

Characteristic	Short delirium	Prolonged delirium	OR (95% CI)	*P* value
	*n* = 22	*n* = 29		
Age*	84.6 ± 4.7	85.6 ± 5.9	1.04 (0.93–1.15)	0.50
Female°	17 (77.3)	21 (72.4)	1.30 (0.36–4.69)	0.69
Mini-Mental State Examination (MMSE) score ^∗¶^	23.0 ± 3.1	19.6 ± 5.5	0.84 (0.71–0.99)	0.02
APACHE II score^∗§^	13.3 ± 3.0	13.9 ± 3.1	1.14 (0.87–1.49)	0.34
Snellen test*	31.6 ± 18.1	38.6 ± 35.5	1.01 (0.98–1.04)	0.41
Barthel ADL Index score^∗△^	17.4 ± 3.0	14.0 ± 4.1	0.76 (0.62–0.93)	0.003
Lawton IADL score^∗*≈*^	14.8 ± 5.8	18.6 ± 8.3	1.08 (0.99–1.17)	0.08
Geriatric Depression Scale-15 score^∗†^	6.4 ± 1.1	6.4 ± 1.7	0.98 (0.61–1.59)	0.95
CRP value*	12.7 ± 25.2	13.4 ± 28.5	1.00 (0.98–1.02)	0.76
History of previous delirium°	0 (0)	12 (46.2)	N.A.	0.001
IQCODE-N > 3.6°	11 (50)	26 (89.7)	8.7 (2.02–37.26)	0.002
Number of concomitant diseases at admission*	2.4 ± 1.5	3.2 ± 2.5	1.20 (0.90–1.60)	0.18
Number of medication at admission*	4.2 ± 2.4	5.6 ± 3.8	1.15 (0.95–1.38)	0.12
MMSE score on the first day of delirium ^∗¶^	18.1 ± 6.3	15.1 ± 6.0	0.92 (0.83–1.03)	0.13
^¥^DRS-R-98 score on the first day of delirium*	18.6 ± 6.4	20.6 ± 6.5	1.05 (0.96–1.15)	0.28

Data are presented as mean ± SD or *n* (%) unless otherwise indicated.

*Continuous variables, °dichotomous variables.

OR: odds ratio, the chance of developing prolonged delirium, CI: confidence interval.

APACHE II: Acute Physiological and Chronic Health Evaluation II.

IQCODE-N: Informant Questionnaire on Cognitive Decline in the Elderly, >3.6 indicates preexistent cognitive decline.

DRS-R-98: Delirium Rating Scale Revised-98.

^¶^Range 0 (severe cognitive impairment) to 30 (no cognitive impairment).

^§^Range 0 (no acute health problems) to 70 (severe acute health problems).

^△^Range 0 (severe disability) to 20 (no disability).

^*≈*^Range 8 (no disability) to 31 (severe disability).

^†^Range 0 (depression not likely) to 15 (depression very likely).

^¥^Range 0 (no delirium symptoms) to 39 (maximum severity).

**Table 2:** Presence of individual DRS-R-98 delirium symptoms on first day of delirium.

DRS-R-98 item	Short delirium (*n* = 22)	Prolonged delirium (*n* = 29)	*P* value
(1) Sleep-wake cycle disturbance	21 (95.5%)	29 (100%)	0.43
(2) Perceptual disturbances and hallucinations	8/21 (38.1%)	13/27 (48.1%)	0.49
(3) Delusions	11/21 (52.4%)	14/27 (51.9%)	0.97
(4) Affective lability	15 (68.2%)	15/28 (53.6%)	0.30
(5) Language problems	18 (81.8%)	22 (75.9%)	0.74
(6) Thought process abnormalities	19 (86.4%)	27 (93.1%)	0.64
(7) Motor agitation	14 (63.6%)	20 (69%)	0.69
(8) Motor retardation	11/21 (52.4%)	20 (69%)	0.23
(9) Orientation problems	22 (100%)	28 (96.6%)	1.00
(10) Attention deficits	22 (100%)	28 (96.6%)	1.00
(11) Short-term memory impairment	20/21 (95.2%)	27/28 (96.4%)	1.00
(12) Long-term memory impairment	12/18 (66.7%)	22/28 (78.6%)	0.37
(13) Visuospatial impairment	9/18 (50%)	21/25 (84%)	0.02

Data are presented as *n* (%) or *n*/*n* (%) in case of missing data.

**Table 3:** Generalised equation estimation (GEE) model for DRS items 1–13 for different lengths of delirium episodes until recovery (count of days). *N* = 113 included observations.

	*β*	SE	df	Wald *χ* ^2^	95% CI	*P*
DRS-R-98 item 6 Thought process abnormalities	−7.9*E* − 6	6.30*E* − 6	1	1.558	−2.03*E* − 5, 4.5*E* − 6	0.21
DRS-R-98 item 9 Orientation	−6*E* − 6	8.88*E* − 6	1	0.456	−2.34*E* − 5, 1.14*E* − 5	0.50
DRS-R-98 item 10 Attention	−5.17*E* − 6	8.83*E* − 6	1	0.343	−2.25*E* − 5, 1.21*E* − 5	0.56
DRS-R-98 item 11 Short-term memory	−1.08*E* − 6	9.67*E* − 6	1	0.012	−2.01*E* − 5, 1.79*E* − 5	0.91
DRS-R-98 item 12 Long-term memory	1.45*E* − 5	7.20*E* − 6	1	4.044	3.67*E* − 7, 2.86*E* − 5	0.04
DRS-R-98 item 13 Visuospatial impairment	8.86*E* − 6	1.16*E* − 5	1	0.580	−1.40*E* − 5, 3.17*E* − 5	0.45
Constant	1.21	0.11	1	119.487	0.99, 1.42	0.000

SE: standard error, C.I.: confidence interval, *E* with a minus sign signals the number of places the decimal point has to be moved to the left.
